# The hypoxic tissue microenvironment as a driver of mucosal inflammatory resolution

**DOI:** 10.3389/fimmu.2023.1124774

**Published:** 2023-01-18

**Authors:** Ian M. Cartwright, Sean P. Colgan

**Affiliations:** ^1^ Division of Gastroenterology and Hepatology, University of Colorado Anschutz Medical Campus, Aurora, CO, United States; ^2^ Department of Medicine, University of Colorado School of Medicine, Aurora, CO, United States; ^3^ Mucosal Inflammation Program, University of Colorado School of Medicine, Aurora, CO, United States; ^4^ Rocky Mountain Regional Veterans Affairs Medical Center, Aurora, CO, United States

**Keywords:** mucosal inflammation, hypoxia, neutrophil, inflammatory resolution, acidification, adenine nucleotides

## Abstract

On the backdrop of all acute inflammatory processes lies the activation of the resolution response. Recent years have witnessed an emerging interest in defining molecular factors that influence the resolution of inflammation. A keystone feature of the mucosal inflammatory microenvironment is hypoxia. The gastrointestinal tract, particularly the colon, exists in a state of physiological hypoxia and during active inflammation, this hypoxic state is enhanced as a result of infiltrating leukocyte oxygen consumption and the activation of oxygen consuming enzymes. Most evidence suggests that mucosal hypoxia promotes the active resolution of inflammation through a variety of mechanisms, including extracellular acidification, purine biosynthesis/salvage, the generation of specialized pro-resolving lipid mediators (ie. resolvins) and altered chemokine/cytokine expression. It is now appreciated that infiltrating innate immune cells (neutrophils, eosinophils, macrophages) have an important role in molding the tissue microenvironment to program an active resolution response. Structural or functional dysregulation of this inflammatory microenvironment can result in the loss of tissue homeostasis and ultimately progression toward chronicity. In this review, we will discuss how inflammatory hypoxia drives mucosal inflammatory resolution and its impact on other microenvironmental factors that influence resolution.

## Introduction

The gastrointestinal (GI) tract is a highly complex tissue lined by an epithelium that covers a surface area of approximately 300m^2^ (1). This mucosal surface has a unique role in regulating contact between the mucosal immune system and the external environment. The GI tract is home to a diverse and densely populated microbiota and the epithelium forms an important barrier which prevents unregulated exposure of luminal antigens with the lamina propria which houses the mucosal immune system (2, 3). In recent years the view of the epithelium as a static barrier has changed. The epithelium harbors intrinsic innate immunity, regulates antigen sensitization, and molds the microbiota (4–6).

It is widely accepted that the GI tract is in a constant state of low-grade inflammation (7). This state of low-grade inflammation is primed by the constant processing of luminal antigenic material and allows for rapid mobilization of the mucosal immune system to antigens and microbes that penetrate the barrier. The inflammatory process is an essential protective response to pathogens, foreign objects, and injury. This response includes increased vascular dilation, changes in capillary permeability, and leukocyte recruitment (8). As seen in other tissues, an essential component of the innate immune response in the GI tract is the recruitment of polymorphonuclear leukocytes (PMN) to the site of infection or injury.REF Along with the intact epithelium, PMN serve at the front line of defense against microbial pathogens. PMN comprise 50-60% of the circulating leukocytes, making them the most abundant leukocyte population in the blood (9, 10). Once in the tissue, PMN perform a variety of antimicrobial functions; including, degranulation and phagocytosis making them a critical component of the bodies first line of defense (11).

To prevent the development of chronic disease, it is essential for the inflammatory process to be highly regulated and that signals to resolve are present so the tissue can return to a healthy state following the inflammatory insult (12). A critical aspect of the resolution response is the clearance of apoptotic PMN, which is completed by monocytes and macrophages (13). PMN apoptosis is an essential component to productive inflammatory resolution. As a point of fact, a major source of tissue damage in inflammatory conditions, including asthma, rheumatoid arthritis, and inflammatory bowel disease (IBD), is attributed to uncleared PMN (14–16). To ensure proper clearance of PMN at sites of acute inflammation, the mammals have evolved complex and highly coordinated process involving anti-inflammatory cytokines, suppression of pro-inflammatory receptors, induction of pro-resolving mediators, and shifts in the inflammatory microenvironment that all benefit a return to homeostasis (17).

It is now well established that inflammatory hypoxia is a direct result of PMN rapid metabolism of O_2_ as the enter the tissue and activate. All tissue compartments are impacted by inflammatory hypoxia, including infiltrating PMN and other immune cells. In recent years there has been a growing appreciation for the role that PMN play in molding the inflammatory microenvironment and that many changes induced by PMN migration prime the microenvironment for resolution (18). PMN infiltration alters extracellular adenine nucleotide concentrations, drives inflammatory acidification, molds chemokine/cytokine signaling, and induces the expression of pro-resolution lipid mediators (19–22). There is a growing body of literature that indicates that effective mucosal responses to inflammation are driven by changes in the tissue microenvironment. Here, we review some of these changes in the inflammatory microenvironment and how resolution of inflammation is influenced by inflammatory hypoxia, which is driven primarily by PMN infiltration (23).

## Hypoxia and oxygen homeostasis

**Figure 1:**
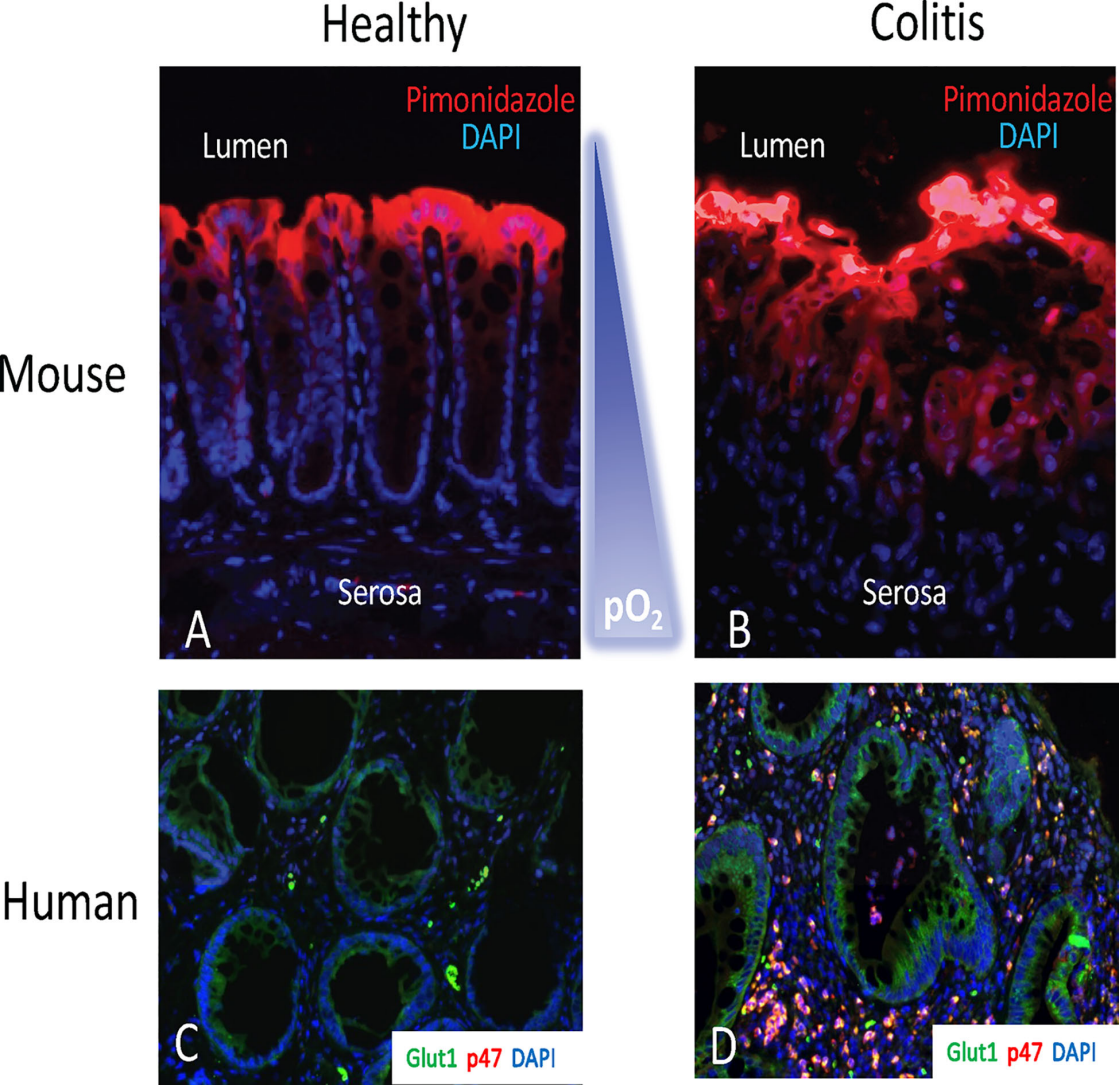
The hypoxic tissue microenvironment in the mucosa. Ulcerative colitis patients with crypt abscesses demonstrate hypoxia-dependent target induction. Panels **(A, B)** depict localization of hypoxic regions using pimonidazole staining (red) with nuclear counterstain (blue) in healthy mouse colon **(A)** and active murine coltis **(B)**. Note the gradient of hypoxia (red) from lumen to serosa. In panels **(C, D)**, colon biopsies from uninflamed margins **(C)** or inflamed regions with active crypt abscess **(D)** in patients with ulcerative colitis were processed for hypoxia-responsive Glut-1 (green), neutrophil p47^phox^ (red) and nuclei (blue).

The gastrointestinal tract presents a unique microenvironment in which there is a steep oxygen gradient between the anaerobic lumen and the highly vascularized and oxygenated subepithelium. At sea level the partial O_2_ pressure (pO_2_) of the air we breathe is ~145 mm Hg and within the alveolus of health lungs the pO_2_ has a range between 100-110 mmHg (24). In stark contrast, the lumen of a healthy intestine exists at a pO_2_ of ~10 mm Hg (25–27). Extensive work using 2-nitroimidazole dyes, a class of compounds which can be utilized to image low-O_2_ environments, has shown that in normal GI mucosa, most notably in the colon, the tissue resides in a state of physiological hypoxia (**Figure 1**). The use of these dyes has revealed that inflammatory lesions in mouse models of colitis show enhanced hypoxia, and even anoxia, and that such hypoxia extends deep into the mucosal tissue (**Figure 1**) (28). This inflammatory hypoxia results from a combination of factors including recruitment of inflammatory cells (PMN, eosinophils, and monocytes), elevated oxidative metabolism, and activation of oxygen-consuming enzymes (29). The use of surrogate markers of hypoxia (e.g. GLUT1) have demonstrated a strong correlation between PMN accumulation and hypoxia in human IBD tissue (**Figure 1**).

Given the physiological hypoxia and near anoxic conditions observed during inflammation, several studies have examined the impact of hypoxia-inducible factor (HIF) stabilization under low O_2_ conditions on epithelial gene expression, specifically promoting epithelial barrier function (27, 30, 31). There are three α isoforms of HIF (HIF-1α, HIF-2α, and HIF-3α). These isoforms are Per-ARNT-Sim (PAS) members of the basic helix-loop-helix (bHLH) family of transcriptions factors (32). Under hypoxic conditions, the α subunit is stabilized in the cytoplasm and following nuclear translocation, the α subunit forms a functional heterodimetric complex with the β subunit HIF-1β (also referred to as aryl hydrocarbon receptor nuclear translocator (ARNT)) (33, 34). In the setting of colitis, HIF-1α has been shown to be essential for regulating the inflammatory process. Epithelial specific loss of HIF-1α results in a significantly more severe colitis then observed in wild-type animals. HIF-1α deficient animals have increased weight loss, decreased colon length, and greatly increased intestinal permeability. In support of these observations, it was also observed that constitutively active intestinal epithelial HIF was protective in murine models of colitis (26). Further studies have demonstrated that HIF-1α stabilization in inflamed tissue promotes the expression of mucins, antimicrobial peptides, and tight junction proteins, all factors that play a pivotal role in limiting inflammation and promoting resolution (35).

PMN have been shown to have an important role in depleting the local O_2_ in inflamed tissue (3). As PMN transmigrate across the epithelium and become activated, they deplete the local O_2_. This depletion of O_2_ is driven by PMN NADPH oxidase and ultimately stabilizes HIF-1α within the epithelium. Studies examining the loss of neutrophil NADPH oxidase and depletion of PMN demonstrated that both the presence of PMN and PMN-induced hypoxia were required for mucosal protection during inflammation and resolution of inflammation (36, 37). The maintenance of barrier function is essential during inflammation and is pivotal to controlling the inflammatory response. In individuals with IBD, marked impairments in tight junction structure and function result in increased epithelial permeability (38). HIF-1α has a critical role in maintaining barrier function and mice deficient in HIF-1α have increase intestinal permeability and barrier function in the context of DSS colitis (26). It has been shown that one of the critical components of the intestinal epithelial tight junction complex, claudin-1, is a HIF-1α transcriptional target, where intestinal epithelial cells lacking HIF-1α manifest significant barrier defects (39). The role of HIF in barrier function during inflammation can also been shown in studies that have stabilized HIF-1α with the use of pharmacological inhibitors targeting polyl hydroxylase (PHD) or genetic deletion of the von Hippel-Lindau gene. In both situations, increased stabilization of HIF-1α results in increased barrier function in the context of colitis (26, 40, 41). Furthermore, HIF-1α stabilization has been demonstrated to induce the expression of several pro-resolution factors, including IL-33 and IL-10, and down regulation of pro-inflammatory cytokines and chemokines in both leukocytes and epithelial cells (42–44). The role of cytokines in the resolution response is discussed later in the review.

## Extracellular adenosine generation and signaling

Another area of considerable interest is PMN-associated adenosine (Ado) signaling in inflammatory resolution (45). It is well established that PMN are a prominent reservoir for Ado precursors and that during inflammation PMN actively release ATP and ADP into the microenvironment (46). More recently, it has been shown that neutrophils are a significant source of diadenosine triphosphate (Ap3A), which is metabolized to ADP by ectonucleotide pyrophosphate/phosphodiesterase-1 (ENPP1) providing an additional source of nucleotides during inflammation (22). It is now widely accepted that ecto-nucleoside triphosphate diphosphohydrolase (NTPDase, CD39) and ecto-5’-nucleotidase (CD73) provide the major pathways for extracellular hydrolysis of ATP and ADP, allowing for the accumulation of extracellular Ado (31, 47, 48). Extracellular Ado can interact with cell surface Ado receptors on the intestinal epithelial cells (49). Currently, four subtypes of G protein-coupled Ado receptors have been identified, AA1R, AA2AR, AA2BR, and AA3R (49).

Several aspects of Ado signaling have been shown to be regulated by hypoxia. As previously discussed, PMN infiltrate is accompanied increases in extracellular adenine nucleotides. It has been shown that enzymes responsible for converting these adenine nucleotides, CD39 and CD73, are regulated by hypoxia. CD39 expression is induced under hypoxic conditions in a SP-1 dependent manner (50, 51). Likewise, CD73 is transcriptionally induced by the direct binding of HIF-1α to the promoter region of CD73, resulting in increased CD73 protein under hypoxic conditions (31, 52). This increase in the potential of intestinal epithelial cells to produce Ado from extracellular ATP and other adenine nucleotides is an important aspect of initiating the resolution response. ATP has been shown to be a proinflammatory molecule and that excessive levels of extracellular ATP can result in systemic inflammatory response syndromes, cytokine storms (53). In addition to enhancing the production of ado, it has also been shown that under hypoxic conditions the expression of the predominant Ado receptor in intestinal epithelial cells, AA2BR, is upregulated. Within the promoter of AA2BR there is a Hypoxia Response Element (HRE) and under hypoxic conditions HIF-1α binds to this site and increases AA2BR transcript expression. Further investigations determined that the increase in transcript was followed by increased protein expression and an overall increase in AA2BR function (54). These increases in Ado generation and signaling capacity are essential for the promotion of resolution in inflamed tissue.

Ado plays a critical role in maintaining tissue barrier function during inflammation. As PMN transmigrate across epithelium there is localized widening of the inter-junctional space which is associated with loosening of the tight junctional protein complex (55). As the PMN move into the luminal space they are actively releasing ATP and ADP, which when convert by epithelial cells into adenosine signals through the AA2BR receptor to stimulate the resealing of the epithelial barrier (56). This resealing is driven by increases in intracellular cyclic AMP (cAMP) following activation of AA2BR by adenosine. The increase in cAMP activates protein kinase A which phosphorylates vasodilator-stimulated phosphoprotein (VASP). Phospho-VASP localizes with ZO-1 and promotes barrier recovery following PMN transmigration (57, 58). Subsequent studies have revealed that VASP mutants with non-functional actin-binding domains results in diminished barrier recovery (58).

In addition to promoting barrier function during inflammation, it has also been reported that adenine nucleotides, including Ado, serve as a primitive defense against bacterial pathogens by promoting a mucosal flushing response. It was first reported *in vitro* that exposure to various adenine nucleotides metabolites induced the apical secretion of Cl- in cultured intestinal epithelial cells (59). These adenine nucleotides are converted to Ado which activates the AA2BR and increases intracellular cyclic adenosine monophosphate (cAMP). This increase in cAMP activates PKA which phosphorylates the cystic fibrosis transmembrane conductance regulator (CFTR) which pumps Cl- into the luminal space, promoting movement of H20 across the epithelial layer (60–62). Within the airway it has been established that loss of CFTR function results in chronic bacterial lung infections (63). Similar observations have been made in the intestines where it was shown that *Escherichia coli* and *Salmonella enteritidis* suppress Cl- secretion and inhibit fluid transport (64, 65). Interestingly, in mice infected with *Salmonella enteritidis* the CFTR was internalized in the crypts of the colon and overall ion transport was decreased, suggesting the observed diarrhea can be attributed to dysfunctions in the epithelial cells ability to absorb H20, not the active secretion of Cl- (65). Furthermore, it has been reported that intestinal epithelial cell specific CFTR promotes the outgrowth of pro-inflammatory bacterial species including *Heliobacter typhlonius* and *Clostridium perfringens* (66).

Finally, it has been shown that intestinal epithelial cells exposed to Ado have marked changes in gene expression. Ado signaling through AA2BR has been demonstrated to elicit pro-resolving responses by acting as an inhibitor of the NF-κB signaling pathway (67) through a number of mechanisms, including elevations in intracellular cyclic adenosine monophosphate (cAMP) (67, 68). More recently it was shown that Ado signaling through AA2BR activates CREB and induces the expression of SLC26A3, a major apical Cl^-^/HCO_3_^-^ exchanger in intestinal epithelial cells. Increased expression of SLC26A3 buffered against PMN transepithelial migration associated acidification. This observation identifies SLC26A3 as an adaptive response in intestinal epithelial cells to maintain pH homeostasis (20).

## Tissue acidification and pH regulation

An often-underappreciated aspect of tissue inflammation is extracellular acidification. Such “inflammatory acidification” (20) has been observed in numerous disease conditions including, cancer, rheumatoid arthritis, and IBD (69). Given the prevalence of inflammatory acidification, it should be considered a hallmark of inflammation. Extracellular acidification impacts cellular signaling and function in a wide range of cell types, including epithelial cells and immune cells (21, 70). At sites of inflammation, it is believed the decrease in extracellular pH is due to the infiltration and activation of inflammatory cells, resulting in an increase in glycolysis and lactic acid secretion (71, 72). Recently, it has been observed that PMN-epithelial interaction results marked inflammatory acidification that was dependent on PMN-epithelial contact, but independent of PMN transepithelial migration. The source of the acidification was identified to be the epithelial cells and is a result of increased lactic acid production and increased ROS generation during PMN-epithelial cell interactions (20, 73).

In addition to PMN-associated acidification, it is widely accepted that tissue hypoxia has a significant impact of tissue acidification. In the absence of cellular O_2,_ the sharp increase in glycolysis results in pyruvate fermentation into lactate, which is transported out of the cell through monocarboxylate transporters (MCT) (74). In this context, lactate has been shown to have both pro- and anti-inflammatory properties (69). Lactate accumulates in chronically inflamed tissue and drives the production of the pro-inflammatory cytokine, IL-17, however, during an acute inflammatory response lactate promotes a resolution response in macrophages but inhibiting the inflammasome (75, 76). In the context of IBD, the role of lactate appears to be cell type-dependent. *In vitro* work demonstrated that overexpression of MCT4 in colonic epithelial cells resulted in of NF-κB activation and translocation to the nucleus which induced IL-6 expression and the dissociation of CREB from the ZO-1 promotor. *In vivo* extensions of the studies revealed that pharmaceutical inhibition of MCT4 increased barrier function in murine models of colitis by decreasing the expression of pro-inflammatory factors and promoting ZO-1 expression (77). Furthermore, It has been shown that there is an increase in the lactate transporter MCT4 and an increase in fecal lactate concentrations (78, 79). However, it has also been shown that loss of GPR81, a G-protein coupled receptor activated by lactate, increases sensitivity to colitis in a dendritic cell dependent pathway (80). There is additional evidence that acute treatment with lactate dampens the immune response by down-regulating glucose uptake and expression of IL-6 (81). In various tissues, it has been shown that lactate is rapidly cleared from tissue into the blood stream and disposed by the liver and kidneys (82, 83). Additionally, when the hypoxic microenvironment resolves, the remaining lactate is converted into pyruvate and utilized for aerobic respiration (84). Taken together, these findings suggest a complex role for lactate in the inflammatory response, where timing and duration of the lactate exposure as well as the ability to clear excess lactate determines the endpoint response.

In a healthy GI tract, a balance between lactic acid-producing and lactic acid-utilizing bacteria are kept low. However, in intestinal dysbiosis (such as IBD) an increase in lactic acid and opportunistic pathogens take advantage of the increase in lactic acid concentrations to out compete other commensals, further driving inflammation (85). Mice lacking lactate dehydrogenase A, the enzyme responsible for converting pyruvate to lactate, results in a significant decrease in inflammation in models of non-infectious colitis (86). Furthermore, it has been observed that mice colonized with lactate-utilizing bacteria experience decreased severity of DSS-induced colitis and that administration of these lactate-utilizing bacteria can reverse the dysbiosis associated with colitis (87).

Independent of lactate, there is a growing body of literature shown that an acidic microenvironment promotes inflammation and suppresses resolution. For example, exposure of PMN to mildly acidic environments (pH 6.5-7.0) results in a significant attenuation of apoptosis, increasing their functional life span. Extracellular acidosis activates the NF-κB pathway and significant accumulation of intracellular cAMP. Under acidic conditions, there is a disruption of the mitochondrial transmembrane potential and translocation of cytochrome c to the cytoplasm, inhibiting apoptosis and prolonging the inflammatory response (88). Furthermore, inflammatory acidification has been shown to negatively impact platelet function at sites of inflammation. Under acidic conditions, platelets show decreases in adhesion, spreading, activation of α_IIb_β_3_ integrin, ATP release, and other functions. Acidic platelets increased neutrophil chemotaxis, activation, and survival, amplifying the neutrophil-mediated inflammatory response (89).

The full impact of inflammatory acidification on intestinal epithelial cell signaling and function remains unclear, however, in recent years there have been several studies that have highlighted the impact of extracellular acidification on epithelial cells. It has been well established that there are several G protein-coupled receptors (GPR 132, GPR4, GPR68, and GPR65), acid-sensitive ion channels (acid-sensing ion channels (ASIC) and transient receptor potential vannilloid-1 (TRPV1) expressed in a wide array of tissues in the body that sense and are activated by increased ion concentrations, the extent of their involvement in sensing pH by intestinal epithelial cells has not been well characterized (90). It was recently shown that intestinal epithelial cells sense extracellular acidosis through GPR31 in a CREB-dependent fashion. Activation of GPR31 by inflammatory acidosis induced an unique gene signature that was observed in both inflamed, acidic murine tissue and in tissue samples from patients with Crohn’s disease (21).

It appears that in the setting of chronic inflammation, the ability of intestinal epithelial cells to balance extracellular pH is diminished. In both murine models of colitis and patient samples from individuals with UC and CD the expression of SLC26A3 is significantly attenuated (20). Epithelial-specific SLC26A3 null mice the mucosal surfaces of the cecum and colon of are significantly more acidic then wild-type controls (91). These epithelial-specific SLC26A3 null mice offer a unique opportunity to investigate acidosis associated changes in the microbiome. It was observed that in SLC26A3 knockout mice there was an outgrowth of *Bacteroidetes* and *Deferribacteres* and a decrease in abundance of *Actinobacteria* and *Firmicutes* (91).

## The role of pro-resolving lipid mediators

Without timely resolution of acute inflammation, chronic disease can develop. In recent years there has been a growing appreciation that specialized pro-resolving mediators (SPM) have a pivotal anti-inflammatory role in the tissue (92). SPM are a large family of mediators and including lipoxins, resolvins, protectins and maresins and are enzymatically derived from essential fatty acids during the acute inflammatory response by both epithelial and immune cells (93–96). SPM are immunoresolvent molecules, which are distinct from immunosuppressive molecules; meaning, they enhance the host defenses while dampening the inflammatory response. SPM signal through G-protein coupled receptors, which include N-formyl peptide receptor 2 (ALX/FPR2), chemokine-like receptor 1 (ChemR23), leukotrience B4 receptor 1 (BLT1) GRP18, and GPR32 and have distinct cell type-specific actions (97–100). For example, in intraepithelial lymphocytes (IEL), GPR18 has been shown to be required for intestinal homeostasis, where mice lacking GPR18 show a dysregulation of CD8 T cells accumulation in the intraepithelial and lamina propria compartment (101). In intestinal epithelial cells ALX/FPR2 and ChemR23 have been shown to be influenced by a number of microenvironmental factors and are important for resolution of inflammation. It has been shown that ALX/FPR2 is induced by Il-13 and interferon γ. Activation of ALX/FPR2 by lipoxins significantly inhibited the production of TNFα and Il-8 by intestinal epithelial cells (102). ChemR23 is highly expressed on apical surface of intestinal epithelial cells and activation by resolvin-E1 induces a wide array of inflammatory regulatory genes including alkaline phosphatase (ALPI). ALPI contributes to the detoxification of bacterial LPS and inhibits bacterial growth (103). In M1 macrophages RvE1 exposure induces the expression of the anti-inflammatory cytokine IL-10 (104). Using multiple murine models of colits, including DSS and TNBS colitis, it has been shown that treatment with both RvE1 and RvD2 improves colitis. RvE1 and RvD2 decrease leukocyte infiltrate and pro-inflammatory cytokines (105, 106). Furthermore, it has been shown that in IBD patients unresponsive to anti-TNFα, ChemR23 expression is increased. A recent study has shown that an agonist anti-ChemR23 antibody promoted resolution of both acute and chronic inflammation in murine models of colitis (107).

The expression of SPMs are highly dependent on the tissue microenvironment. Very quickly following tissue injury or pathogen invasion polyunsaturated fatty acids (PUFA) are released from membrane phospholipids (108). These early PUFA are rapidly metabolized into eicosanoids, which aid in the recruitment of PMN to the site of inflammation. Early in the inflammatory response, PMN recruitment and transmigration triggers lipid mediator class-switching resulting in the metabolism of PUFA into arachidonic acid and ultimately lipoxin, a pro-resolving mediator (109). This shift eicosanoids to lipoxin represses PMN recruitment; while, promoting influx of monocytes to the site of inflammation, stimulating the efferocytosis and promoting resolution and a return to tissue homeostasis (110). Recently, it has been shown that SPM not only impact the recruitment of PMN to site of inflammation, but also have a direct effect of PMN action in the tissue. In a murine *E. coli* lung inflammation model, it has been demonstrated that 15-epi-LAX_4_ and 17-epi-RvD1 signal through the ALX/FPR2 receptor on PMN to inhibit Toll-like receptor 9-mediated release of neutrophil elastase (NE) and proteinase 3 (PR3). NE and PR3 release down-regulates C5aR, resulting in the suppression of PMN phagocytosis and apoptosis, delaying the resolution of inflammation (111). Interestingly, several bacterial species have evolved to utilize SPM to their advantage. *Staphylococcus aureus* release α-hemolysin, which is a potent elicitor of SPM synthesis. This overproduction of SPM dampens the immune response, even in the presence of an ongoing infection (112).

Tissue hypoxia has a significant impact on the production and function of SPM. The influence of hypoxia on the production of SPM was initially observed in endothelial cells, where it was shown that hypoxic endothelial cells upregulate COX-2, an important enzyme in the production of E and D resolvins, and in the presence of Il-1β endothelia produce the precursor to the 17R series resolvin that is enzymatically activated by PMN *via* lipoxygenation and epoxidation. This bioactive 17R series resolvin was shown to inhibit PMN transmigration *in vivo* and diminish zymosan-induced peritonitis (113, 114). When M2 macrophages, the macrophage responsible for clearing apoptotic PMN from inflamed tissue, encounter an hypoxic environment they increase their production of an eicosapentaenoic acid-derived resolvin, RvE4, which promotes the clearance of both apoptotic PMN and senescent red blood cells (115). Another resolvin which strongly inhibits PMN infiltration, RvE2, has also been shown to be induced in PMN by hypoxic conditions (115). In addition to inhibiting PMN infiltration, RvE2 also increases macrophage phagocytosis and production of the anti-inflammatory cytokine, IL-10, promoting overall resolution (116).

## Chemokine/cytokine signaling

Mucosal chemokines and cytokines are well-established in their roles of initiating and driving the inflammatory response; however, until recently their role in the resolution process has been underappreciated. Inflammatory insults are rapidly sensed by epithelial cells and tissue macrophages which triggers the release of numerous proinflammatory cytokines and chemokines (10). The foundation of the resolution response is the cessation of PMN infiltration and induction of apoptosis and eventual clearance of PMN (117). It is now well understood that this process is not passive, i.e dilution of chemokine gradient, but is in fact a highly organized and active process. An important aspect of the resolution response is the production of anti-inflammatory cytokines and chemokines. Prominent anti-inflammatory cytokines include IL-1 receptor antagonist (IL-1ra), IL-4, IL-6, IL-10, IL-11, IL-13, and TGF-β (118, 119). A key feature of these anti-inflammatory cytokines is that they act as immunomodulators that limit excess inflammation. The impact of the anti-inflammatory cytokines dependent on cell type, kinetics of their release, cytokine receptor density, and co-existence of other cytokine and chemokines at the site of inflammation (120).

IL-10 is considered one of the more important anti-inflammatory cytokines. IL-10 is primarily produced by T helper cells, monocytes, and macrophages; however, it has been reported that epithelial cells and granulocytes are also able to produce IL-10 in response to infection or tissue damage (121, 122). IL-10 binds to the heterodimeric IL-10 receptor (IL-10R1 and IL-10R2) which is expressed on a wide array of cell types, including, PMN, macrophages, and epithelial cells (123). It has been shown that IL-10 impacts a wide array of activated PMN functions, including recruitment, LPS-induced cytokine production, and ROS production (123, 124). In recent years it has become evident that IL-10 signaling in epithelial cells in essential for the resolution of inflammation. In response to INFγ inflamed intestinal epithelial cells upregulate IL-10R1 and IL-10 exposure promotes barrier function by repressing the expression of Claudin-2, a claudin often associated with “leaky” epithelial barrier (125, 126). Epithelial specific depletion of IL-10R1 greatly increases DSS colitis severity. Epithelial-specific IL10-R1 null mice have increased intestinal permeability, increased expression of pro-inflammatory cytokines, and a marked decrease in disease resolution (126). As with the other microenvironmental factors discussed in this review, IL-10 expression is influenced by hypoxia. Within B cells, stabilization of HIF under hypoxic conditions results in the induction of IL-10 transcript and secretion of IL-10 into the inflammatory microenvironment which promotes resolution of experimental colitis (44, 127).

Another important cytokine in the resolution response is TGF-β. TGF-β is produced by all leukocytes, epithelial cells, and T cells at sites of inflammation (128). Secreted TGF-β must be activated before it can bind to its receptors and trigger a signaling cascade within the cell. TGF-β can be activated by several extracellular factors, including metalloproteinases and reactive oxygen species (129–131). Within the GI tract, TGF-β suppresses local response to luminal antigens, increases secretion of IgA, and promotes barrier function. Mice lacking TGF-β or TGFβRII develop spontaneous colitis, as well as inflammation in a number of other organs (132–134). In murine models of colitis, it has been shown that induction of TGF-β by a dysregulated microbiota triggers the induction of FGF2, which in turns induces the secretion of IL-17 to promote repair of the epithelial layer and barrier function (135). However, like other anti-inflammatory cytokines, inappropriate activation of TGF-β can result in inappropriate wound healing and can lead to the development of fibrosis or cancers, highlighting the complexity of the resolution response (136).

## Conclusions

**Figure 2:**
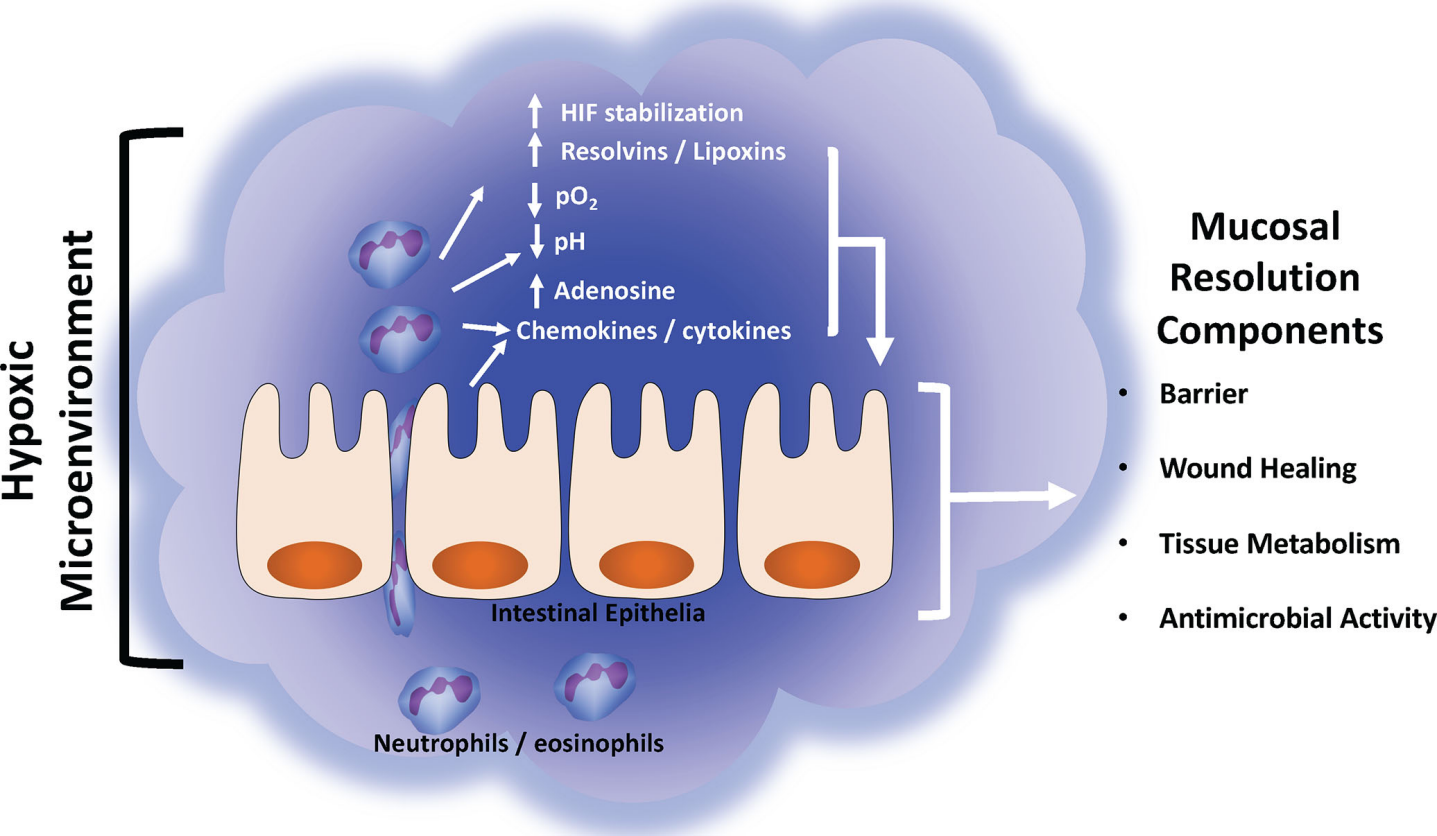
Summary of mucosal inflammatory resolution components driven by the hypoxic tissue microenvironment. During active inflammation, the normally hypoxic microenvironment (physiologic hypoxia) is enhanced (inflammatory hypoxia) by the infiltration and copious consumption local oxygen by innate immune cells (e.g., neutrophils and eosinophils). Such localized hypoxia drives a number of biochemical reactions that enhance the resolution response. See text for details of each resolution component and their respective response to hypoxia.

In this review, we have outlined how various aspects of the mucosal inflammatory microenvironment are influenced by inflammatory hypoxia and how these alterations impact the resolution response. Both *in vitro* and *in vivo* studies have demonstrated that the resolution response is greatly impacted by the extracellular microenvironment and that tissue hypoxia plays a key role in initiating the resolution response by molding the inflammatory microenvironment. As PMN migrate into tissue they deplete the local oxygen and stabilize HIF-1α. This hypoxic environment is associated with increases in both ado generation and expression of Ado receptors, generation of SPM, and secrete anti-inflammatory cytokines, all of which promote the resolution response (**Figure 2**). As highlighted throughout this review, disruptions in the microenvironment can prevent the resolution of acute inflammation and drive the development of a chronic inflammatory lesion. The pharmacologic stabilization of HIF is already showing promise as an anti-inflammatory therapy in human patients (ClinicalTrials.gov: NCT04353791) (137). As highlighted in this review, it has been demonstrated targeting the inflammatory microenvironment can improve colitis in a variety of murine models, however the exact molecular mechanisms and pathways remain elusive. Given that over 50% of individuals with IBD will eventually encounter treatment failure, it is crucial that we indentify novel therapeutic targets (138). Furthering our understanding of how the microenvironment changes during inflammation and how various cell types respond to these changes will provide new insight into potential therapeutic targets for the treatment of human inflammatory diseases such as IBD.

## Author contributions

IC wrote the original draft. SC and IC reviewed and edited the manuscript. All authors contributed to the manuscript and approved the submission.
